# Prevalence of depression and anxiety among school-going adolescents in Indian Kashmir valley during COVID-19 pandemic

**DOI:** 10.1186/s43045-022-00185-1

**Published:** 2022-03-04

**Authors:** Asif Jeelani, Sabira Aalia Dkhar, Ruqia Quansar, S. M. Salim Khan

**Affiliations:** Department of Community Medicine, Government Medical College, Srinagar, Jammu and Kashmir India

**Keywords:** Depression, Anxiety, GAD-7, PHQ-9A, Adolescents

## Abstract

**Background:**

The coronavirus disease 2019 pandemic has led to severe disruption in routine activities, significant mortality and morbidity. Adolescents are particularly prone to mental health issues. The present study aims to estimate prevalence of depression and anxiety and its determinants among school-going adolescents in Kashmir valley of India.

**Results:**

The Patient Health Questionnaire for Adolescents and Generalised Anxiety Disorder questionnaire were used to screen for depression and anxiety among school-going adolescents aged between 15 and 19 years during January and February 2021. Out of the 439 adolescents who had responded, 426 (97.03%) were included in final analysis. The adolescents had a mean age of 17.5 + 1.26 years and comprised of 57% males. The overall prevalence of depression was 16% and was associated with a past history of COVID-19 infection. Anxiety was present in 20% of adolescents. The prevalence was 14% for boys and 27.5% for girls. On logistic regression, anxiety was associated with female gender, past history of personal COVID-19 infection, history of COVID-19 diagnosis in family and hospital admission due to COVID-19 in family.

**Conclusions:**

Anxiety and depression are major public health problems among adolescents. The high burden estimated in our study highlights the need for immediate action to support adolescents particularly those with a self/family history of COVID-19.

## Background

Adolescence is a crucial phase in one’s lifetime as it acts as a bridge between childhood and adulthood [1, 2]. This period is characterised by changes in physical, psychological and social development and is the most common phase in one’s lifetime when mental health issues can emerge [3]. COVID-19 pandemic bought with it multiple lockdowns, social distancing and disease related quarantine. For school-going adolescents, it also led to school closures which may have led to disruption in routine life of adolescents. Schools and colleges hold an important place in the overall growth and development of a child. Social, mental and physical milestones are laid down here, and it the place where the child lays down the foundation for a broader future. Here, the child confronts, learns, interacts and grows which enables them to thrive for future problems [4, 5]. Prolonged school closures also meant that adolescents had to spend more time in their homes [6, 7]. Since peer and other social interactions take more importance during adolescence, it can be expected that lockdowns, social distancing and quarantine associated with COVID-19 may have caused significant disruption in the social life of adolescents [8]. There has been limited literature related to the mental health of adolescents after the onset of the COVID-19 pandemic.

### Aims

The present study was conceptualised to estimate the prevalence of depression and anxiety and its determinants among school-going adolescents in the Kashmir valley of India.

## Methods

### Study design and setting

The study had a cross-sectional study design and was conducted among school-going adolescents with ages ranging from 15 to 19 years (late adolescence). The study was conducted in one administrative unit of district Srinagar of the Kashmir valley. The administrative unit has a total population of around 1 Lakh with an adolescent population of around 17,000. The area is predominantly urban with few pockets of rural and hilly areas.

### Selection of participants

The adolescents were contacted through their school teachers who had the contact numbers of all students as classes were being conducted online owing to pandemic related lockdown. The study area is divided into 12 subcentre areas, and one school was selected randomly from each subcentre area. The teachers of selected schools were contacted, and the link for online questionnaire shared was shared. The teacher then shared the link with the adolescent students. The data was collected online using Google Forms.

### Sample size calculation

Sample size was calculated using the formula for prevalence studies (1.96)^2^ *p*(1-*p*)/L^2^. The prevalence of depression was estimated at 20%. A precision level of 0.01 was used, and the sample required was estimated to be 381 subjects.

### Inclusion criteria

Adolescents aged between 15 and 19 years and studying in the selected schools were included in the study.

### Exclusion criteria

As the questionnaire was shared online with all the students in the eligible age group, no specific exclusion criteria were predetermined.

### Assessment tools and procedure

Data collection was done between January and February 2021. The questionnaire consisted of three parts; the first part contained questions for socio-demographic profile, past personal diagnosis of COVID-19, history of quarantine, history of COVID-19 diagnosis and hospital admission in the family. Self-reported weight, height and duration of physical activity were also recorded. The second part consisted of English version of Patient Health Questionnaire for Adolescents (PHQ-9A), and the third part consisted of English version of Generalised Anxiety Disorder questionnaire (GAD-7). PHQ-9A is a modified version of PHQ-9 to be developmentally appropriate for adolescents. The tool is freely available and consists of nine items. Adolescents are asked to rate the frequency of symptoms they experienced in the past 2 weeks. The responses are recorded on a four-point Likert scale ranging from 0 (not at all) to 3 (nearly every day). The scores of individual items are summed with a maximum possible score of 27. In this study, an elevated score was defined as a total score of 10 or higher. This scale has been validated for use in adolescents. GAD-7 was used to assess the anxiety symptoms. This tool has seven items and asks participants to describe the frequency of each symptom during the last 2 weeks. The responses are recorded on a four-point Likert scale ranging from 0 (not at all) to 3 (nearly every day). Like for the PHQ-9A scale, a score of 10 or more was considered to be positive for anxiety. Since the target population was school-going children and the primary medium of education in this part of the world is English, the English version of the same was used. Face and construct validity of the questionnaire was assessed by independent experts after which the tool pretested on 30 subjects. Cronbach alpha was estimated to be 0.73 which suggested good internal consistency for the questionnaire.

### Main outcome measures

The total scores for both PHQ-9A and GAD-7 were calculated for each subject. A score of 10 or more was labelled as positive for both. BMI was calculated from self-reported weight and height and socioeconomic scale as per the Kuppuswamy scale for 2021 [9].

### Statistical analysis

Means, percentages and quartiles were calculated for a description of the subjects. Fisher exact test and chi-square test were used for univariate data analysis. Variables that had a *p*-value of less than 0.02 were further analysed using logistic regression. Subjects whose responses had more than one missing value were excluded from the study. Data imputation using the average for that specific question was used if there was a single missing response in PHQ-9A or GAD-7 scales.

### Ethical consideration

The study protocols were reviewed by one mental health and one public health expert. Informed consent was sought from all participants and additionally from their guardians as the study population belonged to adolescent’s age group. Approval was obtained in the institutional review committee.

## Results

Out of the 439 adolescents who had responded to any question, 426 (97.03%) were included in final analysis. Of these 426 adolescents, 242 (56.8%) were males. The mean age was 17.5 + 1.26 years. Two hundred fifty-seven (60.4%) had cleared 10th class, and 332 (77.9%) resided in urban areas. As per the latest Kuppuswamy scale, 265 (62.2%) belonged to upper lower and lower middle strata. Mean BMI was 23.1+ 3.1, and mean duration of physical activity was 163.5 + 72 min per week. The study timing corresponded to almost 4 months after the peak of first COVID-19 wave, and in our study, 111 (26.1%) adolescents reported anyone in family getting infected with COVID-19 in the past. In addition, 34 (8%) had a personal positive history for COVID-19 infection, and all these adolescents had been quarantined after the infection. There was a significant increase in the use of the internet from a median of 2 h per day to 4 h per day before and after the start of the pandemic. The details are provided in Table 1.

**Table 1:** Sociodemographic profile of subjects

Variable		*N*	%
Gender	Male	242	56.8
	Female	184	43.2
Age (years)	Mean ± SD	17.48 + 1.26	
Level of education	8th	17	4.0
	9th	59	13.8
	10th	93	21.8
	11th	111	26.1
	12th	146	34.3
Socioeconomic scale	1 (lowest SE class)	68	16.0
	2	143	33.6
	3	122	28.6
	4	68	16.0
	5 (highest SE class)	25	5.9
Physical activity (minutes/week)	Mean ± SD	163.5 (72)	
BMI	Mean ± SD	23.1 + 3.1	
Residence	Urban	332	77.9
	Rural	94	22.1
Personal H/O of any mental illness	Yes	4	0.9
	No	422	99.1
Family diagnosis of any mental illness	Yes	7	1.6
	No	422	99.1
Personal H/O of any chronic illness	Yes	7	1.6
	No	419	98.4
Past history of personal COVID-19 infection	Yes	34	8.0
	No	392	92.0
Past history of COVID-19 infection in family	Yes	111	26.1
	No	315	73.9
Personal history of hospital admission due to COVID-19	Yes	0	0.0
	No	426	100.0
History of hospital admission for any family member due to COVID-19	Yes	34	8.0
	No	392	92.0
History of being quarantined for 14 days	Yes	111	26.1
	No	315	73.9
Impact of COVID-19 on life	1 (Lowest)	34	8.0
	2	155	36.4
	3	161	37.8
	4	59	13.8
	5 (Highest)	17	4.0

We found an overall prevalence of depression to be 16%. The prevalence ranged from 14.5% in adolescent boys to 18% for girls. The mean score for PHQ-9A was 4 with a range of 0–20. The highest mean score was achieved in PHQ-9A scale was attained for question-related to sleep disruption (question no 3), whereas the question related to self-harm had the lowest mean score (question no 9).

The overall prevalence of anxiety was 20% with the prevalence ranging from 14% in boys and 27.5% in girls. The mean score for GAD-7 was 5 with a range of 0–19. For the GAD-7 scale, the highest mean score was achieved for question related to worry, and the least score was for question related to feeling nervous (Table 2).

**Table 2:** Scores achieved on PHQ-9A & GAD-7 and internet use

	Min	Q1	Median	Q3	Max
Depression score (PHQ-9A)	0	3	4	8.5	20
Anxiety score (GAD-7)	0	3	4	8	19
Use of internet before COVID-19	0	2	2	4	6
Use of internet after COVID-19	1	3	4	6	8

Depression assessed by PHQ-9A for adolescents (minimum and maximum scores: 0–27)

Anxiety assessed by GAD-7 (minimum and maximum scores: 0–21)

Being overweight, physical activity of less than 150 min/week, past personal history of COVID-19, history of admission due to COVID-19 in family and history of being put under home quarantine were significantly associated with depression in univariate analysis. Being less than 18 years, female gender, being overweight, rural residence, past personal history of COVID-19 and history of admission due to COVID-19 in family were associated with anxiety in univariate analysis (Table 3).

**Table 3:** Univariate analysis of variables depicting their relation of depression and anxiety

		Total *N* (%)	Depression present *N* (%) Score ≥ 10	*p* value	Anxiety present *N* (%) Score ≥ 10	*p* value
Total		426	68 (15.96)		85 (19.95)	
Age	Less than 18	204	34 (16.67)	0.12	51 (25.00)	0.01
	More than 18	222	34 (15.32)		34 (15.32)	
Gender	Male	242	35 (14.16)		34 (14.05)	
	Female	184	33 (17.93)	0.352	51 (27.72)	0.001
BMI	Underweight	34	2 (5.88)		3 (8.82)	
	Normal	291	40 (13.75)		65 (22.32)	
	Overweight	101	26 (25.74)	0.001	17 (16.83)	0.004
Residence	Urban	332	50 (15.06)		59 (17.77)	
	Rural	94	18 (19.15)	0.34	26 (27.66)	0.04
Physical activity	Less than optimal	186	51 (27.42)		38 (20.43)	
	Optimal and more	240	17 (7.08)	0.00	47 (19.58)	0.82
Level of education	Less than 10th	169	32 (18.93)	0.179	31 (18.34)	0.537
	More than 10th	257	36 (14.01)		54 (21.01)	
Self-diagnosis of any mental illness	Yes	4	4 (100)	0.001	4 (100)	0.007
	No	422	64 (15.17)		81 (19.19)	
Family diagnosis of any mental illness	Yes	7	3 (42.86)	0.13	4 (57.14)	0.044
	No	419	65 (15.51)		81 (19.33)	
Past history of personal COVID-19 infection	Yes	34	26 (76.47)	0.001	22 (64.71)	0.001
	No	392	42 (10.71)		63 (16.07)	
Past history of COVID-19 infection in family	Yes	111	26 (23.42)	0.0158	17 (15.32)	0.169
	No	315	42 (13.33)		68 (21.59)	
History of hospital admission for any family member due to COVID-19	Yes	34	16 (47.06)	0.001	19 (55.88)	0.001
	No	392	52 (13.27)		66 (16.84)	
History of being quarantined for 14 days	Yes	111	26 (23.42)	0.0158	17 (15.32)	0.169
	No	315	42 (13.33)		68 (21.59)	

Fisher exact test used for all cells except for cells with any value less than 5 in which case chi square test with Yates correction used

Numbers in parenthesis are percentages

Correlation of quantitative variables with GAD-7 and PHQ-9A for adolescents

Multivariate logistic regression revealed that history of personal COVID-19 infection was positively associated with depression. Anxiety had a significant relation with female gender, age less than 18 years, history of personal COVID-19 infection, history of COVID-19 infection in family and history of hospital admission due to COVID-19 in the family (Table 4).

**Table 4:** Binary logistic regression of variables for selected variables

		Depression			Anxiety		
		OR adjusted	95% CI	*p* value	OR adjusted	95% CI	*p* value
Gender	Male	1			1		
	Female	1.3	0.83–1.62	0.69	5.3	3.1–7.2	0.00
Age	18 and more	1			1		
	Less than 18	.374	0.12–1.1	0.07	3.1	2.1–5.4	0.06
Residence	Urban	1			1		
	Rural	1.12	0.3–1.8	0..98	1.8	.73	2.2
Physical activity	150 min or more per week	1			1		
	Less than 150 min per week	1.8	0.9–2.5		1.3	0.74–2.2	
BMI	Underweight	1			1		
	Normal	0.42	0.21–1.54		1.2	0.81–1.72	
	overweight	1.2	0.78–1.56	0.98	0.9	0.67–1.53	0.69
Personal past h/o COVID-19	No	1			1		
	Yes	6.3	4.8–7.2	0.0001	6.7	5.1–7.8	0.001
Past h/o COVID-19 in family	No	1			1		
	Yes	1.9	0.7–3.4	0.76	2.1	1.3–3.1	0.002
Hospital admission in family	No	1			1		
	Yes	2.1	1.4–3.7	0.92	3.6	2.5–5.7	0.001
Perceived impact of COVID-19	0–3 on Likert scale	1			1		
	4–5 on scale	1.12	0.73–1.53	0.08	2.6	1.6–4.3	0.001

Binary logistic regression was done for variables that had a *p*-value of less than 0.2 on univariate analysis

## Discussion

We report the results of our study to estimate the prevalence of depression and anxiety among school-going adolescents aged between 15 and 19 years. The study was conducted in January and February 2021, a time period that preceded the deadly second wave of COVID-19 that hit India as well as Kashmir valley. The present study was conducted among school-going adolescents, during a time when the schools had been closed for a year owing to the COVID-19 pandemic.

In this study, the prevalence of depression and anxiety among adolescents was 16% and 20% respectively. Prevalence of depression among adolescents in India ranges from 0.5% to as high as 20% in some studies. The studies have been conducted in varied settings using varied study tools and cut-offs. The overall prevalence is comparable to multiple previous studies conducted in India [10–12]. Studies by Islam MS et al. in Bangladesh and El-Missiry A et al. in Egypt have also found a comparable proportion of adolescents suffering from depression [13, 14]. The prevalence rate of 1 in 7 adolescents observed in our study suggests that depression is a significant public health problem in this part of the world. In the context of sketchy mental health services in the study area, this represents a huge burden on the existing health systems. We estimated the prevalence of depression to be higher in female adolescents (18%) than in adolescent boys (14.5%). The overall prevalence was not statistically significant although mean score on PHQ-9A was significantly higher among female adolescents than their male counterparts. Studies by Mishra SK and Mohta A also estimated higher prevalence of depression among female adolescents [11, 15]. Higher prevalence of depression among females has also been found in developed countries, which suggests that the difference in risk may be determined in part by biological differences between the males and females [16, 17].

Depression was significantly associated with a personal past history of COVID-19 on binary logistic regression. The long-term impact of COVID-19 on physical health has been the subject of intense debate that led to multiple studies being conducted on long COVID-19 syndrome. A previous study by Chen X in China found a significant increase in prevalence of depression during the pandemic [17]. Further studies are warranted among COVID-19 recovered patients to estimate the risk attributed to COVID-19. In addition health systems should screen recovered adolescents for depression so that early counselling and/or treatment may be initiated.

We estimated the prevalence of anxiety to be 19.95% with females having higher prevalence (27.72%) than male adolescents (14.05%). Anxiety was significantly associated with female gender on logistic regression. A previous study by Jayashree K et al. in India also found a higher prevalence of anxiety among female adolescents [18]. Racine N et al. conducted a meta-analysis of studies estimating depression and anxiety among adolescents during the COVID-19. The meta-analysis also found a comparable prevalence of anxiety and also a higher prevalence among females [19]. Like depression, anxiety was also significantly associated with a personal history of COVID-19, but at the same time, anxiety was also associated with a history of COVID-19 in family and a history of COVID-19-associated hospital admission in the family. Pandemic times were very stressful, and the associated home confinement and school closures has the potential of causing increased tendency among adolescents. Adolescents with a self-diagnosis of COVID-19 or a family member diagnosed with COVID-19 were susceptible to social isolation and ostracization, with multiple studies confirming discrimination with COVID-19 patients and their family members [20–22].

Depression and anxiety always remained a significant health problem particularly in adolescence, but the unique challenges posed by this pandemic means that the problem may get graver in coming years. Health systems need to be proactive and scaleup the availability of mental health services particularly in low- and middle-income countries.

In our view, this is the first population-based study among adolescents in this area which we feel is one the primary strengths of this study. Other strengths include the use of validated study tool for both depression and anxiety.

## Conclusions

The present study estimated the prevalence of depression and anxiety to be 16% and 20% respectively. The mean scores were 4 and 5 for PHQ-9A and GAD-7 respectively. Female gender, history of personal COVID-19 infection, hospital admission due to COVID-19 in family and perceived impact of COVID-19 were positively associated with anxiety, whereas history of personal COVID-19 infection was positively associated with depression. The study highlights the need for improving access to preventive and curative mental services for adolescents. There is also a specific need to have a mental health screening program in place for adolescents who had a family history of COVID-19 diagnosis in the family in particular and for all adolescent in general.

### Limitations of the study

We used a validated study tool, but the validity of this study tool has not specifically tested on this study population. Secondly, as online mode was used to disseminate the questionnaire, we could not calculate the non-response rate. The study also employed a purposive sampling method as we did not have a complete sampling frame of all the adolescents in the study area.

## Data Availability

Data are available on reasonable request.

## Abbreviations

PHQ-9 A: Patient Health Questionnaire for adolescents
GAD -7: Generalised Anxiety Disorder questionnaire
